# Poster Session I - A138 FREQUENCY OF ENDOSCOPIC REASSESSMENT FOLLOWING ADVANCED THERAPY INITIATION IN PATIENTS WITH INFLAMMATORY BOWEL DISEASE

**DOI:** 10.1093/jcag/gwaf042.138

**Published:** 2026-02-13

**Authors:** D M Di Fonzo, S Singh

**Affiliations:** Gastroenterology, The University of British Columbia Department of Medicine, Vancouver, BC, Canada; Gastroenterology, The University of British Columbia Department of Medicine, Vancouver, BC, Canada

## Abstract

**Background:**

Both primary and secondary loss of response to therapy is a frequent occurrence in patients with inflammatory bowel disease (IBD). Frequently, practitioners must initiate advanced therapies due to flares of active IBD. Following addition of therapy, it is important to gauge both clinical and endoscopic response.

In 2015, the STRIDE (Selecting Therapeutic Targets in Inflammatory Bowel Disease) working group recommended a shift from clinical definitions of remission to a more stringent endpoint requiring resolution of subclinical inflammation (i.e. endoscopic remission). Specifically, it is recommended to routinely evaluate for endoscopic remission within 6 to 9 months of treatment initiation in patients who have achieved clinical remission but not endoscopic remission, to prevent long-term complications. These recommendations were re-emphasized in a 2020 update (STRIDE-II).

Assessment of endoscopic response is essential, as mucosal healing is an important treatment endpoint which may not always correlate well with clinical symptomology. Ideally, endoscopic reassessment is performed within one year of therapy modifications. However, often endoscopic reassessment may be delayed owing to multiple patient and systemic factors.

**Aims:**

This study aimed to examine the frequency with which endoscopic reassessment is performed within one year of initiation of advanced therapy for IBD.

**Methods:**

This study was performed using retrospective electronic chart review of patients at Vancouver General Hospital (VGH) with a diagnosis of CD or UC. Data was collected on patient demographics, disease phenotype, date of initiation of advanced therapy and date of endoscopic reassessment following initiation of advanced therapy.

**Results:**

Data was collected on a representative sample of 50 patients. Of this sample, 18 patients had a diagnosis of CD, and 32 patients had a diagnosis of UC. 25 patients were on anti-TNF therapy, 14 patients were on anti-integrin therapy, 5 patients were started on anti-IL 23 therapy and 5 patients were on oral small molecule therapy. 17 patients had undergone endoscopic reassessment within one year of initiation of advanced therapy. 15 of the endoscopies were colonoscopies and 2 were flexible sigmoidoscopies. In total, endoscopic reassessment only occurred in 34% of patients within one year following initiation of advanced therapy.

**Conclusions:**

Endoscopic reassessment of intestinal mucosa following initiation of advanced therapy is an important element in optimizing patient outcomes in IBD. In this representative sample, we identified that this practice only occurs in approximately 1/3 of patients following initiation of advanced therapy. Efforts should be made to prioritize more prompt endoscopic reassessment of patients following initiation of advanced therapy. Barriers to this endpoint should be further explored in future studies.

**Funding Agencies:**

None

